# Local and Long-Distance Calling: Conversations between the Gut Microbiota and Intra- and Extra-Gastrointestinal Tract Infections

**DOI:** 10.3389/fcimb.2016.00041

**Published:** 2016-04-11

**Authors:** Joshua E. Denny, Whitney L. Powell, Nathan W. Schmidt

**Affiliations:** Department of Microbiology and Immunology, University of LouisvilleLouisville, KY, USA

**Keywords:** gut microbiome, Infectious Disease Medicine, Cross-talk, influenza, human, *Plasmodium*

## Abstract

Preservation of health from infectious diseases depends upon both mucosal and systemic immunity via the collaborative effort of innate and adaptive immune responses. The proficiency of host immunity stems from robust defense mechanisms—physical barriers and specialized immune cells—and a failure of these mechanisms leads to pathology. Intriguingly, immunocompetence to pathogens can be shaped by the gut microbiome as recent publications highlight a dynamic interplay between the gut microbiome and host susceptibility to infection. Modulation of host immunity to enteric pathogens has long been studied where gut bacteria shape multiple facts of both innate and adaptive immunity. Conversely, the impact of gut commensals on host immunity to extra-gastrointestinal (GI) tract infections has only recently been recognized. In this context, the gut microbiome can augment host immunity to extra-GI tract bacterial, viral, and parasitic pathogens. This review explores the research that affords insight into the role of the gut microbiome in various infectious diseases, with a particular emphasis on extra-GI tract infections. A better understanding of the link between the gut microbiome and infectious disease will be critical for improving global health in the years ahead.

## Introduction

Infectious diseases plague millions of people every year and are the leading cause of death in low-income and developing nations (World Health Organization, 2014; see Box 1). Prevention and treatment of infectious diseases requires a comprehensive understanding of the factors contributing to transmission and risk of infection. Understanding host biological and immunological factors, namely the development, function, and distribution of immune cells, associated with pathogens is important for the development of novel medical interventions and treatments. Yet, we now know the gut microbiome—the collective group of bacteria, viruses, fungi and their associated genes abiding within the intestinal tract—must also be considered as playing a significant role in modulating the host immune response. This microbial community in the gastrointestinal tract is mainly bacterial, containing four main phyla: Bacteroidetes, Firmicutes, Proteobacteria, and Actinobacteria (Eckburg et al., 2005). The composition is relatively stable at the phylum level, but lower taxonomic levels can vary quite widely between two individuals, with differences being shaped by factors such as delivery (i.e., vaginal vs. cesarean), diet and antibiotic use among many others (reviewed by Honda and Littman, 2012; Chevalier et al., 2015; Mikkelsen et al., 2015). It has long been speculated that the number of organisms in the gut is between 10^14^ and 10^15^, resulting in approximately 10-times more microbial cells than human cells; however, a recent calculation determined there are about 10^13^ bacterial cells in humans resulting in the microbe to human ratio closer to 1:1 (Sender et al., 2016). From birth, the gut microbiome contributes greatly to the development of the neonatal immune system as germ-free (GF) mice have underdeveloped immune systems compared to conventionally raised mice (Guarner and Malagelada, 2003). Even more, the gut microbiome influences host health throughout the lifespan as it is linked to various inflammatory and autoimmune conditions, such as irritable bowel syndrome (Hong and Rhee, 2014), inflammatory bowel disease (De Cruz et al., 2012), ulcerative colitis (Flanagan et al., 2011), obesity (Turnbaugh et al., 2006), type 2 diabetes mellitus (van Olden et al., 2015), metabolic syndrome (Vijay-Kumar et al., 2010), and multiple sclerosis (Miyake et al., 2015). The gut microbiome also modulates the immune response to infectious agents both within and outside of the GI tract. In this review we first establish the relationship between the gut flora and the host immune response by discussing several, of the many, intra-GI tract infections affected by the gut microbiota. From there we move into a discussion of the role of the gut microbiome in extra-GI tract infections.

Box 1 **Worldwide burden of infectious disease—does the gut microbiome contribute?**

Among the most prevalent infectious diseases are HIV/AIDS, malaria, and tuberculosis (TB) as these contribute to one-third of deaths in developing nations and territories (Murray et al., 2013). HIV, human immunodeficiency virus, causes significant mortality and morbidity as evidenced by data collected in 2014, which estimated that 1.2 million individuals died due to an HIV infection, or associated illness, and a total of 36.9 million individuals lived with the disease (10 Facts, World Health Organization, 2015). Additionally, nearly 2 million individuals were infected with HIV in 2014, which illustrates the need for improved methods of prevention in communities where HIV transmission is highest, such as sub-Saharan Africa (HIV/AIDS, World Health Organization, 2015). In addition to HIV, malaria is a leading cause of mortality and morbidity when compared to other infectious agents. This parasitic disease is caused by *Plasmodium* spp. and has a global disease burden of 198 million cases and 584,000 deaths across all continents, respectively (World Malaria Report, World Health Organization, 2014). Most of these reported deaths occurred in sub-Saharan Africa in children under the age of 5 years old (World Malaria Report, World Health Organization, 2014). Another infectious disease that currently afflicts two billion individuals across the globe is tuberculosis (TB), caused by the bacterium *Mycobacterium tuberculosis*, with nearly 9 million cases of infection and 1.5 million TB-associated deaths reported in 2013 (Glaziou et al., 2015).

## Interactions between the gut microbiome and gastrointestinal tract infections

### clostridium difficile

*Clostridium difficile* infection (CDI)^1^ is a prominent health care-associated illness that primarily impacts the health of individuals on long-term antibiotic regimens and individuals receiving inpatient care at hospitals or nursing homes (Agha, 2012). Though this gram-positive bacterium is found in the intestinal tract of humans as part of the normal gut microbiota, it can cause disease when the gut microbiota community becomes dysbiotic (Antharam et al., 2013; Koenigsknecht et al., 2015). During dysbiosis *C. difficile* is able to interact with the epithelial cell lining of the gut and promote a pro-inflammatory response that causes numerous clinical symptoms associated with a *C. difficile* infection, such as diarrhea, abdominal pain and sensitivity, low-grade fever, anorexia, and bloody stools (Leber et al., 2015).

The composition of the gut bacterial community is predictive of the degree of *C. difficile* colonization. More specifically, *Porphyromonadaceae, Lachnospiraceae, Lactobacillus, Alistipes*, and *Turicibacter* are associated with colonization resistance, while *Escherichia* and *Streptococcus* are associated with increased susceptibility to *C. difficile* growth (Schubert et al., 2015). Consistent with these observations, colonization of GF mice with *Lachnospiraceae* resulted in a significant decline in *C. difficile* colonization; however GF mice colonized with *E. coli* did not provide resistance to *C. difficile*. These studies demonstrate the ability of certain bacterial species within the gut to inhibit or promote CDI, however there remains much to be learned about the specific gut microbes that promote resistance and susceptibility to CDI. Beyond the bacterial species that inhibit or promote CDI it is also important to determine the physiological mechanisms that explain how differences in the gut community impact susceptibility to CDI as the metabolic profile of the gut microbiota certainly influences susceptibility to CDI (Theriot et al., 2014; Buffie et al., 2015). Antibiotic treatment induces significant changes in gut bacterial populations, which in turn influences metabolite levels (Theriot et al., 2014). Following antibiotic treatment there are changes in the abundance of primary and secondary bile acids, glucose, free fatty acids, dipeptides, and sugar alcohols (Theriot et al., 2014). *C. difficile* exploits these changes and is capable of using metabolites that increase in abundance (e.g., mannitol, fucturse, sorbitol, etc.) as carbon sources for growth. Conversely, certain metabolites confer resistance to CDI. Bacteria such as *Clostridium scindens* that encode the 7α-hydroxysteroid dehydrogenase enzyme, which is needed for secondary bile acid biosynthesis (e.g., deoxycholate and lithocholate) inhibit the growth of *C. difficile* (Buffie et al., 2015).

Though much is still unknown about the relationship between CDI and the gut microbiome, the use of fecal-derived microbiota transplantation (FMT) to treat recurrent CDI has shown great promise in the clinic. For example, FMT has been shown to be well tolerated with an overall clinical cure rate of 89% (Hirsch et al., 2015). 68% of these patients responded to one dose of the orally administered capsules and nearly 21% of patients were cured of CDI following a second dose. In another study, 79% of patients with severe and refractory CDI were cured after intestinal microbiota transplantation (Zainah et al., 2015). These studies highlight the success and future potential of FMT for patients with persistent CDI.

### Citrobacter rodentium

*Citrobacter rodentium* is a gram-negative murine pathogen that colonizes the intestinal mucosa and is used to model enteropathogenic and enterohemorrhagic *E. coli* as well as IBD. *Citrobacter rodentium*, along with pathogenic *E. coli*, depends on attachment and effacement of intestinal epithelial cells for colonization. These pathogens bind to intestinal epithelial cells and disrupt the brush border of the villi while forming an actin pedestal around the attached bacteria, leading to epithelial damage and effacement (Wong et al., 2012). *Citrobacter rodentium* attachment and colonization cause transmissible murine crypt hyperplasia, a form of colitis that induces thickening of the colonic mucosa and inflammation. Ultimately, this inflammation facilitates a loss of diversity and dysbiosis of the gut microbiota (Lupp et al., 2007).

As a gastrointestinal pathogen, *C. rodentium* affects the gut microbiome composition and is in turn affected by the presence or absence of the microbiota, promoting colonization resistance through strategies such as nutrient and niche competition (Lupp et al., 2007). To determine whether specific members of the gut microbiome, or the entire community, are required to prevent *C. rodentium* infection, GF mice were infected with *C. rodentium* followed by colonization with *E. coli, Bacteroides vulgatus*, or *B. thetaiotaomicron*. Colonization with *E. coli*, but neither of the *Bacteroides* species, was effective at outcompeting *C. rodentium* (Kamada et al., 2012). This points to specific commensals as important for resistance to *C. rodentium*, and not just the presence or absence of the gut microbiota as a whole. Additionally, certain gut bacteria like *Lactobacillus plantarum* can exert protective effects on gut integrity by stimulating TLR2 and thus upregulating tight-junction structures, enhancing epithelial barrier functions and reducing overall susceptibility to *C. rodentium* (Karczewski et al., 2010). Resistance to *C. rodentium* through the gut microbiome, as well as resistance to EHEC and EPEC, is also mediated through the presence of segmented filamentous bacteria (SFB), which promote Th17 immune responses in the intestine (Ivanov et al., 2009). During the immune response to *C. rodentium*, interleukin-22 (IL-22) is produced by T helper cells and leads directly to expression of antimicrobial peptides by Paneth cells that help control *C. rodentium*; these peptides include RegIIIβ and RegIIIγ (Zheng et al., 2008), as well as beta-defensins 1, 3, and 4 (Kamada et al., 2012). Toll-like receptors (TLRs) and Nod-like receptors (NLRs) contribute to protection against *C. rodentium* by sensing various microbe-associated molecular patterns (MAMPs) such as LPS and muramyl dipeptide and inducing inflammatory conditions in the gut (Manigold et al., 2000; Khan et al., 2006; Kanneganti et al., 2007). If innate immune sensing is impaired, such as in MyD88-deficient mice, control of *C. rodentium* by neutrophils is lost and bacteremia, colitis, and death ensue (Lebeis et al., 2007). Overall, there is a well-organized response to *C. rodentium* by the host, which is mediated by different members of the gut microbiota.

### Toxoplasma gondii

Toxoplasmosis, an infectious disease caused by the parasite *Toxoplasma gondii*, prevails around the world and an estimated 60 million individuals within the United States are infected with this parasite (2013)^2^. Transmission of *T. gondii* occurs through various routes—congenital transmission, carnivorism, and fecal-oral transmission—with fecal-oral transmission serving as the primary source of widespread infection in the United States and across the globe (2013). Initial control of *T. gondii* infection depends on the efficacy of the mucosal immune response, which includes the collaborative efforts of innate immune defenses, chemokine and antimicrobial secretions, and antigen presentation for activation of the adaptive immune response (Benson et al., 2009). Among these factors, TLR11 is important for detecting the parasite and the production of protective IL-12 and IFN-γ response (Plattner et al., 2008; Yarovinsky et al., 2008).

In some species, including humans, TLR11 is a pseudogene (Roach et al., 2005), yet these hosts still produce a protective IL-12 response by activated dendritic cells (Gazzinelli et al., 1993; Tosh et al., 2016). Recent work has focused on unveiling the TLR11-independent mechanism by which human hosts control *T. gondii* infection (Benson et al., 2009). Protective immunity is, at least in part, attributed to gut bacteria that elicit a TLR11-independent, MyD88-dependent protective immune response following *T. gondii* infection. *T. gondii* infection causes intestinal damage, which results in commensal bacteria indirectly eliciting protective immunity. Following intestinal damage commensal bacteria activate dendritic cells through MyD88-depentent TLRs, primarily TLR2, TLR4, and TLR9, resulting in the production of IL-12 that confers resistance to *T. gondii* (Benson et al., 2009).

## Effect of the gut microbiome on extra-gastrointestinal tract infections and vice versa

In addition to the gut microbiota modulating host immunity to intra-GI tract pathogens, increasing evidence demonstrates its impact on infectious diseases beyond the GI tract (Table 1). Conversely, extra-GI tract infections also appear to impact the composition of the gut microbiota. In the following section we will discuss current literature specifically focused on understanding the role of the gut microbiome in modulating the host immune response to extra-GI tract infections.

**Table 1:** **Extra-gastrointestinal tract infectious diseases modulated by the gut microbiome**.

	**Beneficial gut microbes**	**Pathogenic gut microbes**	**Effect of gut microbiome**	**Immune Structures/Cells**
Influenza virus	*Lactobacillus* (Wang et al., 2014); gram-positive bacteria (Ichinohe et al., 2011)	Segmented filamentous bacteria, *Enterobacteriaceae, E. coli* (Wang et al., 2014)	Promote migration of DCs to lung draining lymph nodes and subsequent T cell priming	Inflammasomes, pulmonary DCs, CD8^+^ T-cells, influenza-specific antibodies, CCR9^+^ CD4^+^ T-cells, IFN-γ, IL-15, T-helper cells (e.g., T_h_17)
LCMV	Not determined	Not determined	Modulate responsiveness to antiviral immune signals (Abt et al., 2012)	Macrophages LCMV-specific CD8+ T cells and antibodies (LCMV-specific IgG)
HIV	Lactobacillales, *Lactobacillus* (Pérez-Santiago et al., 2013), *Bacteroides uniformis* (Mutlu et al., 2014)	Prevotella-rich communities (Lozupone et al., 2014)	Patients with more Lactobacillales showed less intestinal translocation and better response to ART (Pérez-Santiago et al., 2013). Prevotella-rich communities have been associated with numerous pro-inflammatory conditions (Scher et al., 2013)	CD4^+^ T-helper cells (e.g., T_h_17), LPS
*Klebsiella pneumoniae*	Proinflammatory microbes (Fagundes et al., 2012)	IL-10-inducing microbes (Fagundes et al., 2012)	Commensal bacteria promote protective pro-inflammatory cytokines (Fagundes et al., 2012)	IL-10, TLR4, neutrophils
*Mycobacterium tuberculosis*	Not determined	*Lactobacillus* (Winglee et al., 2014), *Helicobacter hepaticus* (Arnold et al., 2015), IL-10-inducing microbes (Arnold et al., 2015)	*Helicobacter hepaticus* is associated with reduced protection against a *Mycobacterium tuberculosis* infection by means of an IL-10-dependent mechanism (Arnold et al., 2015)	CD4^+^ T-helper cells (e.g., T_h_1), TLRs (TLR1 and TLR2), T_h_1 cytokines (e.g., IFN-γ, TNF-α, IL-2, and IL-12), macrophages, granuloma formation
*Plasmodium*	*E. coli* O86:B7 (Yilmaz et al., 2014); *Ruminococcus* (Mooney et al., 2015) Lactobacillaceae, Clostridiaceae (Villarino et al., 2016)	Rikenellaceae, Ruminococcaceae, Bacteroidales, *Turicibacter* (Mooney et al., 2015) Bacteroidaaceae, Prevotellaceae, Sutterellaceae (Villarino et al., 2016)	*E. coli* O86:B7 induces antibodies cross-reactive with *Plasmodium* (Yilmaz et al., 2014); assemblage of bacteria determine severity of malaria (Villarino et al., 2016)	Cross-reactive antibodies; *Plasmodium*-specific antibodies, CD4+ T follicular helper cells, germinal center B cells

### Influenza virus

Flu is an infectious disease in the respiratory tract that is caused by influenza virus (Monto et al., 2000). Infected individuals may develop mild symptoms or severe disease that in some cases leads to death (Monto, 1987). The common symptoms associated with influenza virus include fever, cough, pharyngitis, myalgia, and fatigue (Barik, 2012). Additionally, many patients experience abnormalities in gastrointestinal function, such as vomiting, diarrhea, and nausea, though these symptoms are more common in children than adults (Baden et al., 2009). The seasonal influenza vaccine moderates the clinical burden associated with this virus, but the efficacy of the vaccine depends on the strain of virus dominating the flu season and the number of people vaccinated. A more effective approach for preventing and treating infection warrants an exhaustive understanding of the host-pathogen interaction.

Protection against an influenza infection is associated with the diversity and health of the gut microbiome. Recent findings suggest an important role of gram-positive bacterial populations within the gut and possibly within the nasal tract in fostering an adequate immune response to a respiratory influenza infection (Ichinohe et al., 2011). The gut bacteria provided protection in part by initiating an appropriate inflammatory response through activation of inflammasomes. In the absence of inflammasome activation, lung dendritic cells exhibited reduced migration to the lung draining lymph node resulting in reduced activation of CD8+ T cells. These results correlated with impaired expansion of influenza-specific T cells, influenza-specific antibodies, and elevated viral titers (Ichinohe et al., 2011).

Mice treated with broad-spectrum antibiotics to diminish gut bacteria populations followed by infection with influenza have exacerbated weight loss, a greater drop in blood oxygen saturation, elevated viral titers in the lung, higher epithelial cell necrosis, and more cell death in both the bronchiolar lumen and the bronchiole alveolar lavage (BAL) fluid (Abt et al., 2012). Even more, GF mice experienced significantly more weight loss, a weaker ability to clear influenza, diminished virus-specific antibody titers, and more severe bronchiole epithelial degeneration when compared to outcomes for conventional mice (Abt et al., 2012). Consistent with the work by Ichinohe et al. mice treated with broad-spectrum antibiotics also had fewer influenza-specific antibodies and influenza-specific T cells. Together, these findings suggest a modulatory role of the gut microbiome in eliciting a strong host immune response to influenza.

Gastrointestinal damage that transpires as a result of a respiratory influenza infection is a perplexing phenomenon that is poorly understood. A recent report by Wang et al. demonstrated pronounced changes in the gut bacterial assemblage (2014). Populations that are significantly changed as a result of an influenza infection included a decrease in *Lactobacillus* and increases in both segmented filamentous bacteria (SFB) and *Enterobacteriaceae* (Wang et al., 2014). Influenza-induced gastroenteritis was caused by lung-derived CD4+ T cell-induced dysbiosis that resulted in the expansion of Th17 cells and subsequent intestinal injury. Specifically, during influenza CCR9+ CD4+ T cells are recruited from the lung to intestinal tissues. CD4+ T cell-produced IFN-γ alters the gut microbiota composition, including an increase in *E. coli* that resulted in the production of IL-15. Increased production of IL-15 stimulated *in situ* differentiation of CD4+ T cells into Th17 cells that caused intestinal damage.

### LCMV

Lymphocytic choriomeningitis virus (LCMV) is an Arenavirus that is a naturally occurring rodent pathogen and is often used to study host-pathogen interactions. There are several strains of LCMV that differ in their ability to cause acute vs. chronic infections. Using a strain of LCMV that takes about 1-month to clear post-infection Abt et al. determined how the gut microbiome impacted the host immune response following systemic infection (Abt et al., 2012). Mice treated with antibiotics to deplete the gut microbiome prior to LCMV infection elicited an impaired adaptive immune response to LCMV infection. Specifically, antibiotic treated mice had decreased expansion of LCMV-specific CD8+ T cells, with an elevated expression of inhibitory receptors, and decreased titers of LCMV-specific IgG compared to mice that did not receive antibiotics (Abt et al., 2012). Consistent with these observations, the impaired adaptive immune response in antibiotic treated mice correlated with delayed clearance of LCMV. In addition to changes in the adaptive immune system, this report demonstrated the gut microbiota primes macrophages to respond to interferon stimulation (Abt et al., 2012). Whereas macrophages from antibiotic treated mice have an impaired ability to respond to LCMV infection, the viral titers in antibiotic treated and control mice were similar on day 7 post-infection. Therefore, it is not clear to what effect gut microbiome-dependent priming of macrophages contributes to the delayed clearance of LCMV observed at later time points. One possibility is that the impaired activity of macrophages contributes to the diminished adaptive immune response; alternatively the gut microbiota may modulate adaptive immune responses through another mechanism. Taken together with the previous observations on influenza, the gut microbiome can affect responses to extra-gastrointestinal tract viral infections that have no direct interactions with mucosal surfaces.

### HIV

One of the most prevalent infections impacting global health is human immunodeficiency virus (HIV), which is caused by a retrovirus that invades and infects immune cells of its host, specifically helper T cells, macrophages, and dendritic cells (Kumar et al., 2015). The primary targets of HIV are helper T cells, CD4^+^ T cells, which leads to a progressive loss of these cells over the course of an HIV infection. Eventually, host CD4^+^ T cells drop to a level that cell-mediated immunity of the host ceases, and the likelihood of opportunistic infections substantially increases (Pollock et al., 2015).

Recent observations found that individuals with HIV also have an increased risk for intestinal dysbiosis, culminating in conditions such as diarrhea, colitis, or even metabolic syndrome (Lozupone et al., 2013). In the gut, HIV affects the mucosal immune system by infecting and depleting the Th17 cells in the gut-associated lymphoid tissue (GALT), beginning during the acute phase of infection and continuing throughout the course of the disease (Lozupone et al., 2013). Recent work explored differences among patients with early HIV infection, and the findings ultimately suggested that patients with higher proportions of bacteria from the order Lactobacillales, which contains *Lactobacillus* species, showed lower markers of bacterial translocation, such as LPS in the blood (Pérez-Santiago et al., 2013). Additionally, higher abundance of Lactobacillales in patients was positively associated with CD4+ T cell count and was negatively associated with viral load, indicating that bacteria from Lactobacillales could in some way modulate the infectivity or pathology of HIV infection (Pérez-Santiago et al., 2013). As HIV progresses, translocation of bacteria and bacterial products can occur; for example, LPS can be more readily detected in circulation during HIV infection (Brenchley et al., 2006; Cassol et al., 2010). Translocation during HIV is also associated with higher levels of immune activation and inflammation, along with a poorer response to anti-retroviral therapy (ART) (Brenchley et al., 2006; Cassol et al., 2010). Overall, there is evidence that HIV can induce inflammation and gut permeability during the course of infection, but this pathology can also be partially ameliorated by certain gut bacteria.

### Klebsiella pneumoniae

*Klebsiella pneumoniae* is a gram-negative human commensal and is commonly found as part of the oral and gut microbiota. Whereas *K. pneumoniae* is a normal oral commensal, nosocomial infections in the lower respiratory tract can cause significant diseases, especially in patients that are immunocompromised and those with inadequate respiratory immunity and function (Tsay et al., 2002; Jung et al., 2012). It has been observed that GF mice are more susceptible to pulmonary *K. pneumoniae* infection than conventionally raised mice (Fagundes et al., 2012). Following intratracheal infection with *K. pneumoniae* GF mice produced elevated levels of anti-inflammatory IL-10, reduced neutrophil influx into the lung, and elevated lung bacterial burden and bacteremia compared to control mice. Treatment with TLR ligands, specifically TLR4 stimulation, was sufficient to rescue control of *K. pneumoniae* infection in GF mice. These observations clearly identify a role for commensal flora in shaping the host immune response to pulmonary *K. pneumoniae* infection, however these data do not differentiate the contribution of the lung microbiome vs. the gut microbiome. Indeed, the lung microbiome is involved in other disease states, such as disease progression in cystic fibrosis and asthma (Daniels et al., 2013; Huang et al., 2014).

### Mycobacterium tuberculosis

Tuberculosis (TB) is an infectious disease caused by *Mycobacterium tuberculosis*, an aerobic bacterium that invades the respiratory tract of human hosts. The bacterium primarily establishes infection within the lungs of the host, but the infection can remain latent for the entirety of the host's lifetime as nearly one-third of the world's population has latent TB (Glaziou et al., 2015). Although, extensive research has been conducted on the environmental and immunological factors that contribute to TB, the contribution of the gut microbiome to TB and the effect of TB on the gut microbiome are relatively unexplored. Winglee and colleagues conducted a longitudinal study in a mouse model for TB in order to track changes in the gut microbiome prior to, during, and subsequent to an aerosol infection of *M. tuberculosis* (2014). Overall, significant changes in the resident microbiome occurred between infection and death; in particular, there was a significant loss in gut bacteria diversity initially that was followed by a recovery in diversity (Winglee et al., 2014). Of note, these changes in the gut bacteria community occurred very rapidly, within 6 days after *M. tuberculosis* infection. Given the ability of an influenza infection to modulate gut bacteria populations and intestinal inflammation it would be interesting to know if similar mechanisms are at play following pulmonary TB infection.

### Plasmodium

Malaria is a parasitic infectious disease caused by infection with *Plasmodium* species. In 2013, there were at least 198 million infections and 584,000 deaths attributed to *Plasmodium* infections, with the majority occurring in sub-Saharan Africa (World Malaria Report, World Health Organization, 2014). The life cycle of *Plasmodium* is complex, involving developmental stages in the human host as well as in the insect vector, the *Anopheles* mosquito. Transmission to the human host is initiated when infected mosquitoes deposit sporozoites while taking blood meals; sporozoites enter the bloodstream and migrate to the liver where they infect hepatocytes (Mota et al., 2001; Gueirard et al., 2010). Inside the infected hepatocytes sporozoites differentiate into the asexual merozoites, which are released and establish cyclical infection of red blood cells (RBCs) causing the symptomatic blood stage of infection.

Recent publications have identified links between the gut microbiome and the different developmental stages of *Plasmodium* infections in vertebrate hosts. Beginning with the initial stage of infection, antibodies elicited against glycans present on gut bacteria are able to cross-react with sporozoites. Many microbes, including *Plasmodium*, are able to glycosylate proteins with the Galα1-3Galβ1-4GlcNAc-R glycan (α-gal) (Yilmaz et al., 2014). Importantly, humans do not use that glycosylation pattern and are capable of generating antibody responses directed against α-gal (Yilmaz et al., 2014). The gut pathobiont *Escherichia coli* O86:B7 expresses α-gal that elicits α-gal-specific antibodies that cross-react with α-gal expressed on *Plasmodium* sporozoites (Yilmaz et al., 2014). These antibodies prevent the ability of sporozoites to emigrate from the skin and infect hepatocytes. Consistent with this observation, there was a positive correlation between protection from *Plasmodium falciparum* infection and the presence of α-gal-specific IgM, but not α-gal-specific IgG, in humans (Yilmaz et al., 2014). Of note, the α-gal-specific antibodies did not have any impact on *Plasmodium* infections when sporozoites were injected directly intravenously or when the pre-erythrocytic stage was bypassed and mice were directly infected with parasitized RBCs. These data suggest that certain gastrointestinal bacteria could provide protection against the early stage of the parasite life cycle in humans. Similarly, the composition of the stool bacteria community in Malian children correlated with susceptibility to *P. falciparum* infection, however it did not correlate with progression to febrile malaria (Yooseph et al., 2015). The consistency of this report with that of Yilmaz et al. (susceptibility to sporozoite infection but not parasitized RBC infection) raises the possibility that perhaps Malian children resistant to *P. falciparum* infection harbored higher levels of α-gal-specific IgM titers.

Whereas α-gal-specific antibodies had no effect on the blood stage infection, it has been shown that differences in the composition of the gut microbiome in mice modulates susceptibility to the blood stage infection (Villarino et al., 2016). In this study, C57BL/6 mice from different commercial vendors (Jackson Labs (Jax), Taconic Biosciences (Tac), National Cancer Institute/Charles River Labs (NCI), and Harlan/Envigo (Har)) were infected with *P. yoelii* 17XNL, a rodent-specific *Plasmodium* species. Following infection the severity of malaria was substantially more pronounced in NCI and Har mice compared to Jax and Tac mice. 16S rRNA sequencing from GI samples showed that *P. yoelii*-resistant Jax and Tac mice were enriched for Lactobacillaceae and Clostridiaceae, while *P. yoelii*-susceptible NCI and Har mice were enriched for Bacteroidaceae, Prevotellaceae, and Sutterellaceae. Moreover, colonization of GF mice with cecal content from severe malaria-resistant Jax or-susceptible NCI mice transferred the susceptibility phenotypes, respectively. The report also demonstrated that the gut microbiome could be modulated to decrease susceptibility to severe malaria (Villarino et al., 2016). Examination of the immune response following *P. yoelii* infection showed an increase in the number of germinal center B cells and accelerated antibody class switching in Jax mice compared to NCI mice, which correlated with decreased parasite burden in the Jax mice (Villarino et al., 2016). Taken together, these publications show that the gut microbiome affects *Plasmodium* infections at different points in its life cycle through distinct mechanisms.

Recent reports also demonstrated that the dialog between the gut microbiota and *Plasmodium* is bi-directional. Following infection of mice with *P. yoelii* there is an initial decrease in the alpha diversity and overall abundance of fecal bacteria that returns to pre-infection levels following resolution of the infection (Mooney et al., 2015). The mechanism responsible for this change in gut bacterial composition following *P. yoelii* infection is not known, however it does correlate with sequestration of infected red blood cells in the intestinal vasculature and infiltration of inflammatory immune cells. Similarly, in C57BL/6 mice infected with *Plasmodium berghei* ANKA, which causes experimental cerebral malaria, it has been observed that dysbiosis of the gut microbiota, sequestration of red blood cells in the intestinal vasculature, and intestinal lesions occur during infection, however these differences were not observed in BALB/c mice that are resistant to experimental cerebral malaria (Taniguchi et al., 2015). It remains unclear whether dysbiosis of the gut microbiota observed following *Plasmodium* infection is an effect of the infection, or if the dysbiosis contributes to the severity of malaria. Of note, changes in bacterial diversity have important implications for the role of *Plasmodium* in the pathogenicity of *Salmonella enterica* serotype Typhimurium. *Plasmodium* infections, in both mice and humans, increase susceptibility to invasive bacterial infections, in particular non-typhoid *Salmonella* (Scott et al., 2011; Cunnington et al., 2012; Chau et al., 2013; Mooney et al., 2014). Mooney et al. colonized GF mice with cecal contents from either *P. yoelii*-infected or uninfected control mice followed by *Salmonella* infection; mice that received cecal content from *P. yoelii* infected mice had a higher burden of *Salmonella* in feces than control mice (Mooney et al., 2015). Therefore, not only does *Plasmodium* infection induce dysbiosis of the gut microbiota, but this dysbiosis also leaves the host susceptible to enteric pathogen infections. However, a mechanism for how this occurs has not been elucidated.

## Conclusion

While this review has highlighted recent insight into gut microbiome-dependent control of extra-GI tract infections, there is still much that is unknown. One of the biggest obstacles facing the field is the lack of specific mechanisms that have been elucidated. With the identification of each mechanism, the potential for future therapeutics increases drastically. For example, it has been observed that in mice, short chain fatty acids (SCFAs) such as propionate can circulate to the bone marrow and induce generation of antigen-presenting cells (APCs) that induce diminished Th2 response and thus ablates allergic airway disease (Trompette et al., 2014). An excellent source of SCFAs is fermentation of dietary fiber by the gut microbiome, particularly members of Bacteroidetes and *Bifidobacteria* (Trompette et al., 2014). Thus, uncovering mechanistic interactions between the gut microbiome and the host in the context of extra-GI tract infections should be a top priority.

Although, decades of research have linked the gut flora with susceptibility to enteric pathogens, research in recent years has now demonstrated that the gut microbiota also impact the host immune response to extra-GI tract infections. This effect may not be universal, yet we should not be surprised to learn of other systemic infections that are modulated by gut commensals. It is also becoming increasingly clear that systemic infections can also alter the composition of the gut microbiota. Collectively, these observations point toward novel therapeutic interventions for pathogens, such as *Plasmodium*, that have evaded the development of effective vaccines. Therefore, it is essential we gain a greater understanding of how the gut microbiota communicates with the immune system to affect the outcome of systemic infections.

## Author contributions

All authors listed, have made substantial, direct and intellectual contribution to the work, and approved it for publication.

### Conflict of interest statement

The authors declare that the research was conducted in the absence of any commercial or financial relationships that could be construed as a potential conflict of interest.

## Footnotes

^1^*Clostridium difficile* infection [Online]. Centers for Disease Control and Prevention. Available online at: http://www.cdc.gov/HAI/organisms/cdiff/Cdiff_infect.html

^2^Parasites – Toxoplasmosis (Toxoplasma infection) [Online]. Centers for Disease Control and Prevention. Available online at: http://www.cdc.gov/parasites/toxoplasmosis/gen_info/faqs.html
